# Medical News and Miscellany

**Published:** 1890-02

**Authors:** 


### Medical News and Miscellany

We club with the Cosmopolitan at $3.50 a year. Read the announcement.

THE University of Nashville and Vanderbilt University will hold its annual commencement February 26, inst. We acknowledge the receipt of an invitation—a beautifully executed illuminated card—through the courtesy of Dr. Jos. D. Becton, a graduate; son of ex-President E. P. Becton, of Sulphur Springs, Texas.

REMOVALS.—Dr. I. H. Brewton, from Center Point to Floresville.

Dr. C. E. Kellar, from New Berlin to Luling.

Dr. T. J. Pugh, from Hearne to New Birmingham.

Dr. Sam I. Fox, from Plantersville to Willis.

Dr. E. R. Walker, from Sublime to Schulenburg.

Dr. L. W. Cocke, late of San Marcos, and more recently of Boerne, has removed to Laredo. He has organized the Rio Grande Medical Society, and reforms have been instituted.

MARRIED.—January 29th ult., Dr. W. H. Seale, of Marqueze, Texas, to Miss Beulah Johnson, of Centre Point.

Dr. Geo. Mott and Miss Josie Jordan, at Kountze, Texas, November 25, 1889.

Dr. R. W. Noble, of Barclay, Texas, to Miss Eunice Little, of Temple. December 19, 1889.

THE BEST OPENING in East Texas for an Active Physician.—For sale: A good home, and a practice, worth $3000 a year. Good dwelling with all necessary accessories; one hundred acres rich land; thirty acres in a high state of cultivation; good water. Two churches, postoffice, good schools, etc. A bargain, liberal terms. Address: A. T. Perkins, M. D., Patroon, Shelby county, Texas.

Small-pox in Texas.—Quarantine Officer A. S. Wolff, of the Brazos-Santiago Station, is at Rio Grande City, where the small-pox has broken out in an aggravated form. The doctor had vaccinated four hundred people, and says the work is not one-fourth done. He hopes to be able to suppress the epidemic soon, but says some of the ranches are perfect pest-houses. A singular fact, and one that makes much against his success, is that Mexicans will bring their children hundreds of miles in order to expose them to infection, saying that as "everybody must have small-pox, it is better to have it in childhood." Dr. Wolff will make a final report to the Journal when the work is finished.

---

Died—At his home, Tyler, Texas, January 20, ultimo, Dr. U. G. M. Walker, aged 53 years. Dr. Walker was a native of Alabama, and was born in Dallas county, in that State, March 4, 1836. He was a graduate of the University of Nashville, class of 1857. During the war, he was Surgeon of Flournoy's regiment; later he was a member of the Board of Medical Examiners of Walker's Division, and still later Chief Surgeon of the Division. He served in the Texas Legislature in 1872. The physicians of Tyler held a meeting and passed resolutions of regret at his death, and testified to the high esteem in which the departed brother had always been held. Dr. Q. A. Shuford presided at the meeting.

---

Sorry He Said It.—Mr. J. H. Chambers, publisher, of St. Louis, wrote the Journal a sharp and witty letter on receipt of the January number. He didn't like our comments on his letter to the medical press, and said so. In that letter among other ridiculous things, he said: "The Annals has no use for the general medical journals." He authorizes us now to say that that utterance was indiscreet, and that it was not intended by any means to say that the Annals held the other journals as worthless, but that they were not *necessary* to the Annals. But for our "free ad." he says he has placed our "worthy journal upon the Annals of Surgery exchange list." Thanks; small favors, etc.

FLOWERS.—The flower catalogue of V. H. Hallock & Son, of Queens, New York, is certainly "a thing of beauty," and if not "a joy forever," its perusal gives great pleasure to flower lovers, at least for a season. We have carefully read it, and can truthfuliy say that, quality considered, their prices for plants, bulbs and seeds are surprisingly low. The house caters especially to the Texas trade, and is offering extraordinary inducements to Texas buyers in the way of plants especially suited to this climate. By the by—a circular sent out by this firm is embelished with a picture of Sam Houston, and—by way of anti-climax or antithesis or anti-something—one of Tom Ochiltree! Houston is the Texas idol,—but Tom Ochiltree?—Well, Hallock meant it for a joke, we presume, or to typify his mammoth "Big Sunflower," or—an idea strikes us—they meant Tom for a type of the "lilies of the field"—which "toil not;" and as to purple and fine linen, Solomon in all his glory was never gotten up so regardless—nor held such hands at poker. Send for Hallock's Floral Catalogue.

---

DR. YOAKUM upon presenting a paper to his medical society, prefaced his remarks with the following flight, which we think should be preserved as a warning, for he was elected President just afterwards:

"GENTLEMEN:—You have often heard of the far-farmed bird of freedom with wings expanded from the 'heads of the rivers to the ends of the earth'; whose fan-like tail cools the face of the silver-veiled queen of night, and whose ear-splitting scream rouses old Morpheus from his opium pipe and shakes the shingles upon the wood-shed of Epluribus Unum. You have also read of the bulls of Bashan, whose fiery eyes burnt holes in the shirt of Kingdom Come, and blistered the cuticle of his Osinominta; and whose elongated and active narrative, jerking itself into knots at every stroke, swept the flies from the cradle of Orion, and the cigar stumps from the streets of Fiddler's Green. Neither are you ignorant of the gigantic and multitudinous achievements in the important sphere of materia medica, a sphere whose rapid and violent revolutions have never yet failed to raise the wind; which wind, when once up, like Bancho's ghost, is never permanently downed, but which wafts prosperity to the habitation of the doctor to a greater or less extent. Of all these you have heard, read or felt; but I come before you now with a series of recent remedies, the very mention of whose effects can not fail to elevate the hair upon your learned parietals and open your optics like a muscle with the ague."

At this point the Association collapsed, our reporter fainted and was carried to the nearest saloon and revived with a glass of ice water.—Exchange.

---

PERSONALS.—Dr. A. B. Gardner, of Bellville, is at the New York Polyclinic, and will take a three months course. He directs the JOURNAL to be sent to him there.

Dr. P. K. Wortham, of Meridian, Bosque county, has removed to Waco.

Dr. O. M. Conerly, whose removal to Bevins, was announced to take place January 1st, has concluded not to move; his address is as before, Wayne, Cass county.

Vice President I. E. Clarke, T. S. M. A., of Shulenburg, is attending the New York Polyclinic; will be back in time to "take in" the big meeting at Fort Worth in April.

Dr. O. L. Williams has removed from Chapel Hill to Oak Cliffs, Dallas.

Dr. John H. Pope, once President of Texas State Medical Association, is located at Lithia Springs, Ga., and makes a specialty of nervous diseases, and treatment of opium and other bad habits.

Dr. C. M. Rosser has retired from the co-editorship of the Courier Record,—a big loss to that journal. He has associated himself with Dr. J. S. Letcher in practice in Dallas. The Doctor appreciates DANIEL'S TEXAS MEDICAL JOURNAL, and getting it no longer as an exchange sends in his subscription, pledging his support; all of which is very gratifying. See the Doctor's letter in this number.

Dr. Wm. Osborn, of Axtelle, whose place was advertised for sale, in the last number of the JOURNAL, has found a purchaser, and has removed to New Birmingham, Rusk county. Dr. Rogers, of New Birmingham, has removed to Axtelle.

